# Socioeconomic Status is Significantly Associated with Dietary Salt Intakes and Blood Pressure in Japanese Workers (J-HOPE Study)

**DOI:** 10.3390/ijerph10030980

**Published:** 2013-03-11

**Authors:** Koichi Miyaki, Yixuan Song, Setsuko Taneichi, Akizumi Tsutsumi, Hideki Hashimoto, Norito Kawakami, Masaya Takahashi, Akihito Shimazu, Akiomi Inoue, Sumiko Kurioka, Takuro Shimbo

**Affiliations:** 1 Division of Clinical Epidemiology, Department of Clinical Research and Informatics, National Center for Global Health and Medicine, Toyama 1-21-1, Shinjuku-ku, Tokyo 162-8655, Japan; E-Mails: isyuson@ri.ncgm.go.jp (Y.S.); taneichi.setsuko@mail.u-tokyo.ac.jp (S.T.); tshimbo@hosp.ncgm.go.jp (T.S.); 2 Office for Mental Health Support, Division for Counseling and Support, the University of Tokyo, 7-3-1 Hongo, Bunkyo-ku, Tokyo 113-0033, Japan; 3 Department of Public Health, Kitasato University School of Medicine, 1-15-1 Kitasato, Minami-ku, Sagamihara 252-0373, Japan; E-Mail: akizumi@kitasato-u.ac.jp; 4 Department of Health Economics and Epidemiology Research, School of Public Health, University of Tokyo, 7-3-1 Hongo, Bunkyo-ku, Tokyo 113-0033, Japan; E-Mail: hidehashimoto-circ@umin.ac.jp; 5 Department of Mental Health, Tokyo University Graduate School of Medicine, 7-3-1 Hongo, Bunkyo-ku, Tokyo 113-0033, Japan; E-Mails: norito@m.u-tokyo.ac.jp (N.K.); ashimazu@m.u-tokyo.ac.jp (A.S.); 6 National Institute of Occupational Safety and Health, Nagao 6-21-1, Tama-Ku, Kawasaki 214-8585, Japan; E-Mail: takaham@h.jniosh.go.jp; 7 Department of Mental Health, Institute of Industrial Ecological Sciences, University of Occupational and Environmental Health, 1-1, Iseigaoka, Yahata-nishi-ku, Kitakyushu 807-8555, Japan; E-Mail: akiomi-tky@umin.ac.jp; 8 Department of Health Policy and Management, University of Occupational and Environmental Health, 1-1, Iseigaoka, Yahata-nishi-ku, Kitakyushu 807-8555, Japan; E-Mail: kurioka@med.uoeh-u.ac.jp

**Keywords:** socioeconomic status, salt intake, blood pressure, hypertension

## Abstract

The association of socioeconomic status (SES) with nutrients intakes attracts public attention worldwide. In the current study, we examined the associations of SES with dietary salt intake and health outcomes in general Japanese workers (2,266) who participated in this Japanese occupational cohort. SES was assessed by a self-administered questionnaire. Dietary intakes were assessed with a validated, brief, self-administered diet history questionnaire (BDHQ). Multiple linear regression and stratified analysis were used to evaluate the associations of salt intake with the confounding factors. Education levels and household incomes were significantly associated with salt intake, as well as blood pressures (*P* < 0.05). After adjusting for age, sex and total energy intake, both years of education and household income significantly affect the salt intake (for education, β = −0.031, *P* = 0.040; for household income, β = −0.046, *P* = 0.003). SES factors also affect the risk of hypertension, those subjects with higher levels of education or income had lower risk to become hypertensive (ORs for education was 0.904, *P* < 0.001; ORs for income was 0.956, *P* = 0.032). Our results show that SES is an independent determinant of salt intake and blood pressure, in order to lower the risk of hypertension, the efforts to narrow the social status gaps should be considered by the health policy-makers.

## 1. Introduction

The association of socioeconomic status (SES) with health outcomes has been well documented: the poorer the SES the worse the prospects for health development [1]. In England, an association between socioeconomic position and mortality was found by the Whitehall II study [2]. Persons with disadvantaged SES suffer from higher rates of some common diseases, such as abdominal obesity [3], metabolic syndrome [4], hypertension [5,6], diabetes [7], atherosclerosis [8] and cardiovascular disease (CVD) [9]. These lifestyle diseases are considered to have a direct relation with nutrition and diet. The social inequities in health may be partially explained by different dietary habits [10,11,12]. 

During the past century, the evidence for the risks imposed on human health by excess salt consumption has become compelling. The association between habitual dietary salt intake and blood pressure has been established through experimental, epidemiological and intervention studies [13]. A meta-analysis confirmed that high salt intake is associated with significantly increased risk of stroke and total CVD [14]. A Cochrane Review focusing on the effects of sodium reduction confirmed that the sodium reduction resulted in significant decrease in blood pressure in either normotensives or hypertensives, as well as increase in plasma renin, aldosterone, adrenaline, noradrenaline, cholesterol and triglyceride [15]. However, the association between salt metabolism and health outcomes is yet controversial. In a prospective study involving 3,681 European participants [16], although systolic blood pressure increases with the increase of urinary sodium excretion, lower sodium excretion was suggested to be associated higher CVD mortality. In another large-scale cohort study of Canada, the association between sodium excretion and cardiovascular events was J-shaped: increasing risks of cardiovascular events were found in both persons with quite high or quite low sodium excretion [17]. 

Murakami *et al.* suggested that neighborhood socioeconomic disadvantage was associated with higher ratio of 24 h urinary sodium to potassium in young Japanese women [18]. In a Brazilian study about homemade complementary foods for infants, the portion of samples which had excessive sodium content were more frequently found in those prepared for infants in low SES families [19]. However, compared with other nutrients such as vitamins or minerals, the studies focusing on the relationship of salt intake with SES factors, especially for the education and income level, is few. In the current study, our objective was to reveal the associations between SES factors, dietary salt intake and blood pressures in our occupational cohort. 

## 2. Materials and Methods

### 2.1. Subjects

The present cross-sectional study was performed as a part of the Japanese study of Health, Occupation and Psychosocial factors related Equity (J-HOPE), which aimed to develop and expand research to elucidate mechanisms underlying the social disparity in health and establishment of measures to control over it. It was based on a baseline survey of our occupational cohort study on social class and health, supported by a grant from the Ministry of Education, Culture, Sports, Science and Technology, Japan. A total of 14,534 individuals from 13 independent cohorts enrolled in this study. In the current investigation, employees of a Japanese major manufacturing company (Headquarter is in Kyoto and the other major 21 offices were spread all over the Japan) were recruited. Approximately 2,500 workers of this company were invited to participate, and 2,266 agreed (response rate 90.1%). The protocol and explanation documents of our study were approved by the ethics committee of the University of Tokyo School of Medicine, and written informed consent was obtained from each subject. 

### 2.2. Measurements

Age, sex, height, weight, systolic and diastolic blood pressures (SBP and DBP), fasting plasma glucose level (FPG), serum lipid levels (total cholesterol, triglyceride, high density lipoprotein (HDL) cholesterol) were measured at health check-ups in all subjects. Body mass index (BMI) was calculated as dividing the weight (in kilograms) by the square of the height (in meters). Blood pressure was determined twice by well-trained nurses and using a form PWV/ABI device (Nippon Colin, Aichi, Japan) with the subjects at rest in a supine position. This device was approved by the US Food and Drug Administration (FDA) as VP-2000/1000. The blood pressure of each subject was calculated as the mean of two values, then, the subjects with a SBP greater than 140 mmHg and/or a DBP greater than 90 mmHg were considered to have hypertension.

### 2.3. Socioeconomic Status (SES)

Years of education, annual household income added by income of each family member, and the numbers of family members were assessed by self-administered questionnaire. Each participant was asked to answer that his/her household income belongs to which of six income grades defined as: 1, <3.0 million yen/year; 2, 3.0–4.99 million yen/year; 3, 5.0–7.99 million yen/year; 4, 8.0–9.99 million yen/year; 5, 10.0–15.0 million yen/year; 6, ＞15.0 million yen/year. The midpoint of each grade was calculated (1, 1.5 million yen/year; 2, 4.0 million yen/year; 3, 6.5 million yen/year; 4, 9.0 million yen/year; 5, 12.5 million yen/year; 6, 20.0 million yen/year). The classification of education subgroups is based on the International Standard Classification of Education (ISCED) (approved by the United Nations Educational Scientific and Cultural Organization (UNESCO) General Conference at its 29th session in November 1997): “Low” education level corresponds to the end of compulsory education, and can also include vocational training after schooling (less than 12 years, ISCED level 1 and 2); “Middle” education level corresponds to at least 3 years of additional schooling, which includes programs designed to provide access to higher education or leads directly to the labor market (12–15 years, ISCED level 3 and 4); “High” education level corresponds to a Bachelor’s degree or higher (≥16 years, ISCED level 5 and 6). 

### 2.4. Dietary Intake

Dietary habits during the preceding month were assessed with a validated, brief, self-administered diet history questionnaire (BDHQ) [20]. Responses to the BDHQ were checked for completeness and, where necessary, clarified by direct questioning of the subject. The BDHQ is a 4-page structured questionnaire that enquires about the consumption frequency of a total of 56 food and beverage items, with specified serving sizes described in terms of the natural portion or the standard weight and volume measurement of servings commonly consumed in general Japanese populations. The BDHQ was developed based on a comprehensive (16-page) version of a validated self-administered diet history questionnaire [21,22,23]. Intakes of table salt and salt-containing seasoning at the table, such as soya sauce and soup consumed with noodles, were estimated from answers to the corresponding qualitative questions. The validation of the BDHQ was performed by using 16-day weighed dietary records as the gold standard, and a weak correlation was observed both for sodium and potassium after energy and creatinine adjustment in men (the adjusted Pearson correlation coefficients of sodium were 0.35 and 0.25 in men and women) [22]. Adjusted salt intakes were calculated as daily salt intakes divided by daily total energy intakes (1,000 kJ). 

### 2.5. Statistical Analysis

Descriptive statistics of clinical characteristics among different education or income groups were compared. Pearson’s correlation coefficients and *P* values represent the relationships between SES factors and intake levels. The association between salt intake and SES factors was examined by multiple linear regression analyses, controlling for age, sex and total energy intakes. The total subjects were stratified into SES subgroups, we calculated age-, sex- and total energy intake-adjusted intake level for salt of each subject, and compared the mean adjusted values between subgroups by using Bonferroni-corrected trend test. Multiple linear regression and logistic regression analysis were used to evaluate the association of SES factors with blood pressures or prevalence of hypertension. The IBM SPSS statistics for Windows version 19.0 J (IBM, Armonk, NY, USA) statistics software packages were used for all statistical analyses. Statistical significance for all analyses was defined as *p* < 0.05. 

## 3. Results

Table 1, Table 2 illustrate the basic characteristics, SES factors and intake levels of the participants by education (Table 1) or income level (Table 2). The mean (±standard deviation, SD) age and BMI of the total subjects (n = 2,266) were 43.4 ± 9.8 years (ranged from 21 to 65 years) and 23.1 ± 3.3 kg/m^2 ^(ranged from 13.8 to 41.8 kg/m^2^), respectively, which are typical for middle-aged Japanese population. 241 of them are women, accounting for 10.6%. Only one third of the total subjects meet the Japanese RDA of salt intake (<9.0 g/day for men and <7.5 g/day for women, n = 753, 33.2%) [24]. 

The correlations of two major SES factors, education and household income, with dietary salt intakes were evaluated and the results are present in Table 3. Both years of education and household incomes negatively related to salt intakes, Pearson’s correlation coefficients (R) were −0.129 (*P* < 0.001) and −0.043 (*P* = 0.042), respectively. 

When the subjects were classified into subgroups according to either education or income levels, age and sex ratio were significantly different by SES subgroups (Table 1, Table 2, *P* < 0.001). Among education subgroups, blood pressures (SBP and DBP) and the dietary intake levels of salt after adjustment by total energy intake, were found to linearly decrease with the increase in education levels (standardized regression coefficients (β) were −0.129, −0.163 and −0.037, respectively) after adjusting for age and sex. As to the income subgroups, there were significant positive associations in BMI (β = 0.045), and negative associations in SBP and energy-adjusted salt intakes (β were −0.056 and −0.119, respectively).

**Figure 1:**
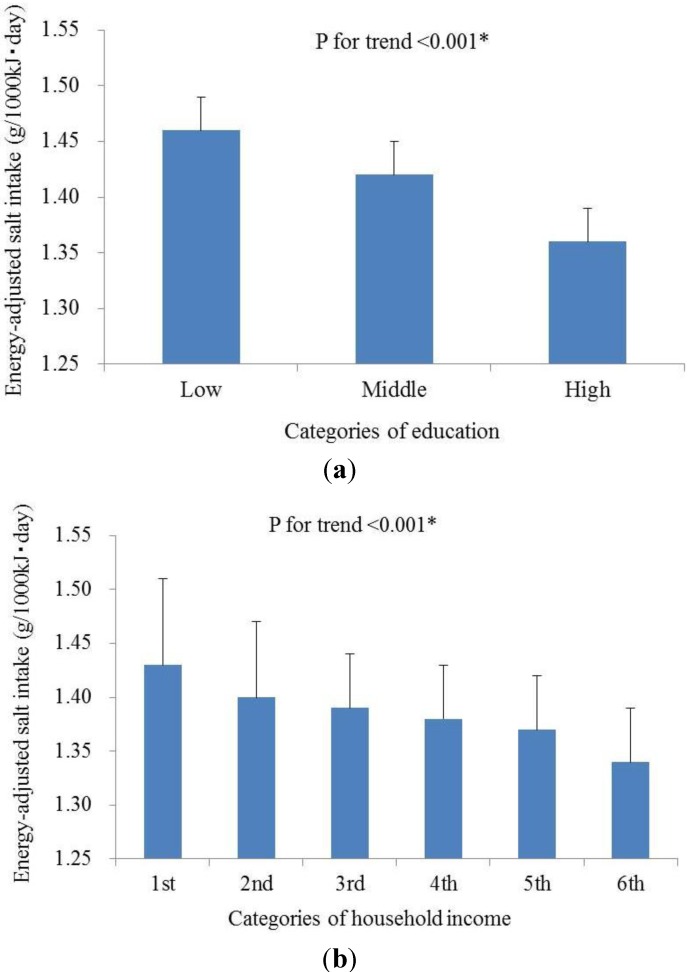
The associations of (**a**) education levels classified by the International Standard Classification of Education (ISCED) and (**b**) household incomes with daily salt intake levels. The classification of education subgroups is based on the International Standard Classification of Education (ISCED), approved by the United Nations Educational Scientific and Cultural Organization (UNESCO). Six subgroups are classified according to the self-reported household incomes of participants: 1, <3.0 million yen/year; 2, 3.0–4.99 million yen/year; 3, 5.0–7.99 million yen/year; 4, 8.0–9.99 million yen/year; 5, 10.0–15.0 million yen/year; 6, ＞15.0 million yen/year. Mean values of energy-adjusted salt intake and standard errors are present. (**a**) Salt-education; (**b**) Salt-income.

In a trend test in which SES factors were used as categorized variables, the age-, sex- and total energy intake-adjusted intake salt level of each subject was calculated, and the mean adjusted values were compared between subgroups. The age, sex and total energy intake adjusted salt intake was significantly associated with both years of education and household income: the intake level decreased with the increasing of education (Figure 1(a), *P* for trend< 0.001) or income (Figure 1(b), *P* for trend< 0.001). 

We next assessed the effects of SES factors on intake levels by a multiple linear regression model. In this analysis, education level and annual household income were added at the same time, and age, sex and total energy intake were used as confounding factors. The results are shown in Table 3. Elder age, male gender, higher energy intake, poorer education level and lower income were significantly associated with higher salt intake. P value for education and income were 0.040 and 0.003, respectively (Table 3). Considering the different dietary habits between genders, we also carried out the separate analysis in male and female subjects. In male subjects accounting for nearly 90% of the total, the results were similar to those of all subjects, the association of education levels with salt intakes was weakened as P value for trend was 0.053 (β = −0.032), meanwhile the association of household incomes with salt intake remained (β = −0.049, P = 0.003). As to the women, neither the education nor the income was associated with the salt intake levels (data not shown).

**Table 1:** Clinical characteristics, dietary nutrients intake data, and socioeconomical status factors of the study subjects according to different education level groups.

	Total subjects (n = 2,266)	Low education level group (n = 131)	Middle education level group (n = 943)	High education level group (n = 1,192)	*P* for trend adjusted for age and sex
Age (year)	43.4 ± 9.8	51.6 ± 9.7	45.6 ± 9.0	40.9 ± 9.4	<0.001 **
Proportion of women (%)	10.6	5.3	16.3	6.7	<0.001 **
Clinical characteristics					
Body mass index (kg/m^2^)	23.1 ± 3.3	23.3 ± 3.7	23.0 ± 3.4	23.1 ± 3.1	0.287
Systolic blood pressure (mmHg)	123.4 ± 16.1	130.8 ± 19.1	125.0 ± 16.2	121.2 ± 15.2	<0.001 **
Diastolic blood pressure (mmHg)	77.1 ± 12.0	81.4 ± 12.5	78.6 ± 11.9	75.4 ± 11.6	<0.001 **
Serum total cholesterol (mg/dL)	200.0 ± 35.1	209.0 ± 34.0	199.2 ± 33.5	199.6 ± 36.6	0.831
Serum triglyceride (mg/dL)	125.8 ± 180.0	142.4 ± 109.9	120.5 ± 89.7	128.8 ± 242.4	0.977
Serum HDL cholesterol (mg/dL)	61.8 ± 16.5	59.8 ± 17.0	62.9 ± 17.1	61.0 ± 15.8	0.679
Fasting plasma glucose (mg/dL)	95.0 ± 23.2	100.7 ± 25.8	95.4 ± 25.3	94.2 ± 20.6	0.245
SES factors					
Years of education (year)	14.5 ± 2.5	9.4 ± 0.7	12.6 ± 0.9	16.7 ± 1.0	<0.001 **
Managerial Position (%)	22.7	10.7	9.4	34.6	<0.001 **
Annual household income (ten thousands yen/year)	704.4 ± 297.5	656.9 ± 336.4	665.9 ± 255.9	740.1 ± 318.6	<0.001 **
Dietary intake levels					
Total energy intake (kJ/day)	7,705.8 ± 2,396.0	7,527.6 ± 2,560.4	7,423.5 ± 2,333.0	7,948.6 ± 2,402.3	<0.001 **
Total energy adjusted salt intake (g/1,000 kJ·day)	1.39 ± 0.31	1.46 ± 0.35	1.42 ± 0.33	1.36 ± 0.28	<0.001 **

Values are shown as mean ± standard deviation or percentage. The classification of education subgroups is based on the International Standard Classification of Education (ISCED) 1997. Subjects with low (<12 years), middle (12–15 years) and high (≥16 years) levels of education were compared. For continuous variables we used linear regression analysis adjusted for age and sex. For categorized variables, we used logistic regression analysis adjusted for age and sex. * *P* < 0.05, ** *P* < 0.01.

**Table 2:** Clinical characteristics, dietary nutrients intake data, and socioeconomical status factors of the study subjects according to different household income groups.

	Total subjects (n = 2,266)	Group 1 (n = 76)	Group 2 (n = 472)	Group 3 (n = 1,049)	Group 4 (n = 403)	Group 5 (n = 239)	Group 6 (n = 26)	*P* for trend adjusted for age and sex
Age (year)	43.4 ± 9.8	34.5 ± 13.5	36.6 ± 11.0	44.0 ± 7.7	48.6 ± 7.3	48.4 ± 7.7	48.9 ± 7.7	<0.001 **
Proportion of women (%)	10.6	31.6	13.8	7.0	7.7	18.0	15.4	<0.001 **
Clinical characteristics								
Body mass index (kg/m^2^)	23.1 ± 3.3	21.8 ± 3.5	22.7 ± 3.4	23.3 ± 3.3	23.1 ± 3.0	23.3 ± 2.9	24.6 ± 3.6	0.049 *
Systolic blood pressure (mmHg)	123.4 ± 16.1	124.0 ± 20.4	122.1 ± 15.7	123.6 ± 16.1	124.2 ± 16.1	123.4 ± 15.8	127.2 ± 17.3	0.013 *
Diastolic blood pressure (mmHg)	77.1 ± 12.0	73.0 ± 12.4	74.3 ± 11.6	77.8 ± 11.9	78.4 ± 11.5	77.6 ± 12.6	79.3 ± 13.7	0.547
Serum total cholesterol (mg/dL)	200.0 ± 35.1	186.4 ± 26.1	193.3 ± 36.0	200.9 ± 36.5	202.4 ± 31.5	201.9 ± 33.0	199.6 ± 34.5	0.132
Serum triglyceride (mg/dL)	125.8 ± 180.0	100.0 ± 51.3	116.3 ± 85.6	130.2 ± 230.1	123.5 ± 94.1	120.5 ± 130.5	166.0 ± 149.3	0.866
Serum HDL cholesterol (mg/dL)	61.8 ± 16.5	60.4 ± 13.3	62.3 ± 16.3	61.6 ± 16.7	61.3 ± 16.3	63.6 ± 16.7	58.7 ± 16.4	0.931
Fasting plasma glucose (mg/dL)	95.0 ± 23.2	89.5 ± 23.4	92.2 ± 21.8	95.2 ± 23.6	97.1 ± 22.2	93.7 ± 20.2	103.2 ± 45.8	0.954
SES factors								
Years of education (year)	14.5 ± 2.5	14.1 ± 2.9	14.6 ± 2.7	14.3 ± 2.6	14.8 ± 2.3	15.2 ± 2.0	15.6 ± 3.5	<0.001 **
Managerial Position (%)	22.7	0	2.5	13.1	50.9	60.3	65.4	<0.001 **
Annual household income (ten thousands yen/year)	443.0 ± 188.5	127.5 ± 29.3	312.2 ± 87.1	415.6 ± 123.2	522.5 ± 129.3	720.5 ± 151.5	1078.3 ± 167.6	<0.001 **
Dietary intake levels								
Total energy intake (kJ/day)	7,705.8 ± 2,396.0	7,424.5 ± 2,763.3	7,496.8 ± 2,461.2	7,780.5 ± 2,427.8	7,827.8 ± 2,209.2	7,717.7 ± 2,310.5	7,389.8 ± 2,196.8	0.022 *
Total energy adjusted salt intake (g/1,000 kJ·day)	1.39 ± 0.31	1.43 ± 0.35	1.40 ± 0.31	1.39 ± 0.32	1.38 ± 0.29	1.37 ± 0.26	1.34 ± 0.36	<0.001 **

Values are shown as mean ± standard deviation or percentage. Subjects are classified into 6 grades according to their self-reported household incomes (1, <3.0 million yen/year; 2, 3.0–4.99 million yen/year; 3, 5.0–7.99 million yen/year; 4, 8.0–9.99 million yen/year; 5, 10.0–15.0 million yen/year; 6, >15.0 million yen/year) and are compared among different grades. The income data for one person is missing. For continuous variables we used linear regression analysis adjusted for age and sex. For categorized variables, we used logistic regression analysis adjusted for age and sex. * *P* < 0.05, ** *P* < 0.01.

**Table 3:** The associations of daily salt intakes with SES factors (years of education and adjusted annual household income).

	Relations of SES factors with total energy adjusted salt intakes		Associations of salt intakes with multiple factors	
	Pearson’s correlation coefficient (R)	P value	Standardized regression coefficient (β)	*P* value
Constant				<0.001 **
Years of education (year)	−0.129	<0.001 **	−0.031	0.040 *
Annual household income (million yen/year)	−0.043	0.042 *	−0.046	0.003 **
Age (years)			0.082	<0.001 **
Sex (male = 1, female = 2)			−0.059	<0.001 **
Total energy intake (kJ/day)			0.753	<0.001 **

P values and Pearson’s correlation Coefficients between intake levels and SES factors, or P values and β (Standardized regression coefficient) showing the significance for linear regression analysis are present. * *P* < 0.05; ** *P* < 0.01.

We also investigated whether the SES factors affect the salt-related health outcomes. As the result, education level was negatively associated with both blood pressures with consideration of confounding factors of age, sex and BMI (SBP: β = −0.128, *P* < 0.001; DBP: β = −0.093, *P* < 0.001), and the household income had a weaker association with SBP only (SBP: β = −0.053, *P* = 0.014; DBP: β = −0.021, *P* = 0.335). In addition, both of the two SES factors influenced the risk of becoming hypertensive: the higher of the education level or the household income, the lesser prevalence of hypertension (years of education: ORs (95% CI): 0.904 (0.863–0.947), *P* < 0.001; household income: ORs (95% CI): 0.956 (0.918–0.996), *P* = 0.032). It is worth noting that when the two SES factors were added into the analysis model at the same time, all of the effects on blood pressures or prevalence of hypertension of household income disappeared (Table 4), meanwhile the associations of education remained. As to the relations between salt intake level and blood pressure, we found that the salt intake was significantly associated with both SBP and DBP (SBP: β = 0.059, *P* = 0.007; DBP: β = 0.064, *P* = 0.003), however, when the age and sex were added as adjusted factors, the significance disappeared (SBP: β = 0.009, *P* = 0.689; DBP: β = 0.011, *P* = 0.598). If the SES factors, education and income were added at the same time, the results were similar to those without consideration of SES.

**Table 4:** The associations of SES factors with blood pressures (SBP and DBP) and the prevalence of hypertension.

	SBP		DBP		Prevalence of hypertension	
	Standardized regression coefficient (β)	P value	Standardized regression coefficient (β)	P value	ORs (95% CI)	*P* value
Years of education (year)	−0.123	<0.001 **	−0.095	<0.001 **	0.911 (0.867–0.956)	<0.001 **
Annual household income (million yen/year)	−0.017	0.458	0.008	0.728	0.980 (0.939–1.022)	0.346
Age (year)	0.155	<0.001 **	0.235	<0.001 **	1.049 (1.034–1.063)	<0.001 **
Sex (male = 1, female = 2)	−0.145	<0.001 **	−0.129	<0.001 **	0.414 (0.244–0.702)	0.001
BMI (kg/m^2^)	0.220	<0.001 **	0.215	<0.001 **	1.135 (1.098–1.173)	<0.001 **

P values and β (Standardized regression coefficient) showing the significance for linear regression analysis, or Odds Ratios and 95% Confidence interval and P values obtained from the multiple logistic regression analysis are present. * *P* < 0.05; ** *P* < 0.01.

## 4. Discussion

In the current study, we confirmed that SES factors, education level and/or household income, were associated with the dietary salt intakes of Japanese workers. It is well known that the sodium intake was very high in Japan, especially before 1950s and early 1960s. Although the public health campaigns in Japan to lower sodium intake made a fall successfully [25], the mean daily salt intake reported by the National Nutrition Survey was 10.7 g per capita per day [26], meeting neither the old (<10.0 g/day for men and <8.0 g/day for women) nor the new (<9.0 g/day for men and <7.5 g/day for women) Japanese RDA of salt intake. In our data the mean (±SD) salt intake was 10.4 ± 3.2 g/day, two thirds (n = 1,512) take excessive salt, indicating the large room for nutritional intervention to relieve the SES effect on health outcomes.

We found that the dietary salt intake showed statistically significant and linear associations with both years of education and household income (Figure 1) in Japanese workers. In previous studies it was reported that persons from socioeconomically disadvantages backgrounds were less likely to purchase foods with low salt [27] or have engaged in salt restriction [28], and our results is agreement with that the intakes of total sodium, salt and soy sauce decreased as educational level increased in Chinese population [29].

Although some studies have shown the correlations between high blood pressure or high prevalence of hypertension and low SES in different population groups, such as Swiss [30], American [31], French [32], Israel [6], Chinese [33] or Thai [34], other epidemiologic studies with no association [35,36] or conflicting results [37,38] were also reported. Our observation provided new evidence supporting the negative association between SES and blood pressure or prevalence of hypertension by using our Japanese occupational cohort. Markedly, our results show the importance of education. After adjusting for age, sex and BMI, education level was significantly associated with SBP, DBP and prevalence of hypertension (Table 4). However, household income levels were not associated with these outcomes. Although the individual associations of household incomes were significant, when it was added together with education, its effects were masked. It suggested that these two SES factors correlate to each other and the influence on blood pressure of education is stronger. In due consideration of the fact that raising income levels dramatically is quite difficult, education may have a realistic power to relieve the SES effect on health outcomes. Our results in general workers are consistent with them and revealed the importance of raising the education level on people’s health.

Some limitations of the present study are worth mentioning. First, the dietary data were obtained from a self-administered semi-quantitative dietary assessment questionnaire [21] in which the actual dietary habits were not observed. Although the validity of this questionnaire appears reasonable [23], the results should be interpreted with caution. Second, our subjects were workers only in a company and not representative for general Japanese population, however, the workers were recruited from 22 offices all over Japan (From Hokkaido to Kyushu). So the geographical deviation was reasonably diluted, but it is noteworthy fact that our result is the result of a large company, not a small one. Finally, we could not obtain some important information, such as the smoking status, alcohol drinking and whether were in treatment for chronic diseases, these issues should be addressed in future study. 

## 5. Conclusions

In conclusion, education and income were strong and independent predictors of salt intake level in general Japanese workers. Our large-scale cross-sectional study indicated the importance of education level in the determination of blood pressures. For lowering the risk of hypertension within a population group, the efforts to narrow the social status gaps may also indirectly contribute to the solution of the important healthy problems.
